# The Fenestras Elisabeth Complex (Nova Structura) in the Parietal Bone of *Plecotus auritus*: Morphology, Topography, and Functional Significance

**DOI:** 10.3390/ani16010109

**Published:** 2025-12-30

**Authors:** Grzegorz Kłys, Paweł Socha

**Affiliations:** 1 Institute of Biology, Opole University, Oleska Street 22, 45-052 Opole, Poland; 2 Laboratory of Microcomputed Tomography, Department of Palaeozoology, Faculty of Biological Sciences, University of Wrocław, Sienkiewicza Street 21, 55-335 Wrocław, Poland; pawel.socha@uwr.edu.pl

**Keywords:** echolocation, Chiroptera, *Plecotus auritus*, parietal bone, cranial fenestration, micro-CT, acoustic adaptation, structural variability

## Abstract

Fenestrations of mammalian skull bones are rare and poorly understood, particularly within the parietal bone. In bats—a group characterised by advanced sensory specialisation and echolocation—superficial modifications of the cranial vault may have functional significance, yet their occurrence and organisation remain insufficiently documented. In this study, we describe an exceptional fenestrated complex in the posterolateral parietal bone of *Plecotus auritus*, comprising structural elements not previously recorded within Vespertilionidae. The aim of the study was to characterise in detail the morphology, topography, and variability of the surface structure termed Fenestras Elisabeth (nova structura), with particular emphasis on its relationship with the auditory region and its potential biomechanical–acoustic significance. The material consisted of ten skulls of *P. auritus*, examined using micro-CT scanning, 3D reconstruction, and qualitative analysis of fenestrated structures and their topographic relationships. Within the posterolateral parietal region, we identified an extensive and repeatable fenestrated complex comprising numerous fenestrae parietales Elisabeth, paired fenestrula Elisabeth, a central depression (recessus acousticus parietalis), and a bordering fissure (fissura occipitalis mastoidea, nova structura topographica). The complex exhibited a stable spatial organisation despite individual variation in the number and shape of the openings. All fenestrations were confined to the posterolateral zone, and the contact between the fenestrae and the fissura occipitalis mastoidea represented a diagnostic feature. Our analysis suggests that the Fenestras Elisabeth complex may be associated with combined biomechanical and acoustic constraints: (1) a biomechanical role—reducing strain in the parietal lamina during movements of the extremely mobile and elongated pinnae; and (2) an acoustic role—modulating micro-vibrations transmitted to the inner-ear structures. Individual variability and lateral asymmetry of fenestration patterns indicate a high degree of adaptive plasticity in this region of the skull. This study provides the first documentation of a large, structurally organised parietal-bone fenestration complex in *Plecotus auritus*, expanding current knowledge of bat cranial morphology and suggesting a likely functional significance for these previously unknown bony structures.

## Simple Summary

The skulls of mammals are shaped by a balance between mechanical stability, sensory function, and weight reduction. In bats, which rely heavily on echolocation and highly mobile external ears, even small structural modifications of the skull may have important functional consequences. In this study, we describe a previously unrecognized fenestrated complex in the posterolateral region of the parietal bone of the brown long-eared bat (*Plecotus auritus*), which we name the Fenestras Elisabeth complex. Using micro-computed tomography, we show that this complex consists of multiple small openings arranged in a repeatable, species-specific pattern, together with a paired set of larger fenestrations and a centrally located depression. Although the number and shape of individual openings vary between specimens, the overall topographic organization of the complex is consistent across all examined skulls. We suggest that this fenestrated architecture may contribute to local lightening of the skull while maintaining structural integrity, and may also play a role in redistributing mechanical stresses and microvibrations associated with ear movements during echolocation. Our findings highlight how subtle skeletal modifications can reflect functional and evolutionary adaptations in bats.

## 1. Introduction

The mammalian skull exhibits substantial morphological diversity reflecting adaptations to a wide range of biomechanical and sensory functions. In bats (Chiroptera), cranial structures play an important protective role while also participating in the precise reception and analysis of acoustic stimuli, forming a key component of the echolocation system [1,2]. Previous morphological studies have primarily focused on elements associated with dentition, mastication, the orbital region, or the basicranium, whereas surface modifications of the cranial vault have remained comparatively understudied.

*Plecotus auritus* (brown long-eared bat) is characterized by extremely elongated and highly mobile pinnae, which play a crucial role in passive listening and precise sound localisation during echolocation. The exceptional mobility and size of the pinnae impose specific biomechanical and acoustic demands on the cranial vault, particularly in the posterolateral region of the skull. These functional constraints provide an important biological context for interpreting structural modifications of the parietal bone observed in this species.

In several bat taxa, such as *Corynorhinus* or *Nyctophilus*, local parietal-bone perforations have been described; however, these structures were few in number, irregular in shape, and did not form a repeatable pattern [2,3]. Species belonging to the genus *Plecotus* are characterised by extremely enlarged and mobile pinnae, which play a crucial role in directing acoustic waves and precisely localising sound sources [4]. With the advent of high-resolution micro-CT techniques, it has become possible to detect subtle cranial features with potential functional or taxonomic importance, including surface fenestration, laminar thinning, and variation in suture trajectories [5,6]. Morphological variability of the skull may additionally reflect environmental or developmental factors influencing the sensory–functional organisation of the head [7].

In *Plecotus auritus*, we identified an extensive and consistently organised suite of novel morphological features in the posterolateral region of the parietal bone, collectively forming a fenestrated complex termed Fenestras Elisabeth (nova structura). This complex includes a topographic fissure (fissura occipitalis mastoidea, nova structura topographica) and paired fenestrula Elisabeth, situated around a central depression—the recessus acousticus parietalis. Together, these elements create a spatially coherent system with potential sensory and functional significance not previously documented in Vespertilionidae.

Unlike the irregular and spatially limited parietal perforations described in *Pteropus* [2], *Myotis* [5], or *Noctilio leporinus* [8], the fenestrations in *Plecotus auritus* form an extensive, highly organised, and repeatable pattern, clearly distinct from the previously described localised and inconsistent modifications.

The aim of this study was to describe and analyse in detail the morphology, topography and variability of the Fenestras Elisabeth surface using micro-CT data and 3D reconstructions, and to determine its structural characteristics and topographic relationships within the posterolateral portion of the parietal bone of *Plecotus auritus*. The names fenestra Elisabeth and the associated units of this complex were given in honour of Elżbieta Koenig—the master’s student of the first author and the initiator of research on bat cranial morphology. Due to its unique fenestrated structure and consistent posterolateral location within the parietal bone, this surface represents a newly defined morphological unit identified during the present study.

## 2. Materials and Methods

### 2.1. Material

The study was based on 10 skulls of the bat *Plecotus auritus* (Linnaeus, 1758) originating from the reference collections of the University of Opole and the Institute of Systematics and Evolution of Animals, Polish Academy of Sciences (ISEZ PAN), Kraków. All specimens were in good osteological condition and suitable for micro-CT analysis and detailed three-dimensional reconstruction.

A detailed list of specimens is as follows:

1. University of Opole—Stobrawski Landscape Park, Poland.
2. University of Opole—Opole, Poland.
3. University of Opole—Tarnowskie Góry, Poland.
4. ISEZ PAN—M/III90, Poland.
5. ISEZ PAN—17.02.1972, Złoty Stok, Poland.
6. ISEZ PAN—M/11194, Solna Jama, Gniewoszów, Poland.
7. ISEZ PAN—05.04.1963, Zbójecka Cave, Ojców, Poland.
8. ISEZ PAN—M/1631/60, Hungary.
9. ISEZ PAN—M/1631/60, Radochowska Cave near Lądek-Zdrój, Poland.
10. ISEZ PAN—M/2450/62, Bielany, Kraków, Poland.

All examined specimens represented skeletally mature (adult) individuals, as indicated by complete dentition and fully ossified cranial elements; no juvenile/subadult skulls were included. Sex information was not available for all museum specimens and therefore was not used as a grouping variable. Only intact, undamaged skulls without visible mechanical damage in the parietal/occipital region were selected for analysis.

The skulls were examined as dry museum specimens and were not subjected by the authors to additional chemical treatment (e.g., fixation, staining, or degreasing) prior to scanning. The analyzed fenestrated area shows a repeatable topographic arrangement across specimens, which is inconsistent with random preparation-related mechanical damage.

Basic morphometric measurements (lengths, widths, proportions, and topographic relationships) were carried out in Dragonfly 2024.1, with a measurement accuracy of 0.01 mm. Measurements were performed using the built-in measurement tools in Dragonfly (v2024.1). Where applicable, each linear measurement was taken three times by the same observer and the mean value was recorded to reduce random measurement error. These measurements were intended to support descriptive characterization and were not used for hypothesis-testing statistics in the present study. A comprehensive morphometric analysis—including variation within Fenestras Elisabeth (nova structura)—will be presented in a separate study.

All skulls are kept in the reference collections of ISEZ PAN in Kraków and at the Natural History Collections Centre of the Institute of Biology of the University of Opole. Sample numbers and catalogues containing voxel data and reconstruction parameters were organised and preserved in the project archive, ensuring full reproducibility of all analyses and enabling re-evaluation upon request from editors or reviewers.

### 2.2. Micro-CT Scanning

Micro-CT scans were acquired at the Microtomography Laboratory of the University of Wrocław, using a Skyscan 1273 system (Bruker).

Acquisition parameters were as follows:

- X-ray tube voltage: 36–41 kV.
- Current: 114 μA.
- Exposure time: 2360 ms.
- Total scan duration: 2 h 6 min.
- Number of projections/averaging: 10.
- Rotation range: 180°.
- Rotation step: 0.4°.
- Voxel size: 9.2 μm (reconstruction matrix 3072 × 1944 px).
- Filter: none.
- Flat-field correction: on.
- Ring-artifact reduction: on.

Reconstruction settings (including ring-artifact reduction and beam-hardening correction) were applied using standard manufacturer-recommended procedures and were visually validated by inspecting representative slices for residual artefacts and preservation of fine cortical details in the parietal region.

Scanning parameters were optimised to maximise the quality of thin-walled and fenestrated structures characteristic of the cranial vault as well as of the basicranium of *Plecotus auritus*.

### 2.3. Data Reconstruction

Projection data were reconstructed using NRecon (Bruker, v.2) with standard reconstruction settings, including:

- beam-hardening correction;
- filtering and smoothing;
- flat-field correction (FF);
- ring-artifact reduction.

Reconstructed datasets were exported as 16-bit TIFF stacks, preserving the full intensity range and geometry of the specimens.

### 2.4. Digital Processing and Visualisation

Micro-CT reconstructions were further processed for:

- generating axial, sagittal, and coronal sections;
- segmentation of bony structures;
- 3D reconstruction;
- morphological analysis of thin-walled elements (nova structura);
- atlas-style documentation of designated structures.

Visualisation and image preparation were performed using:

- Dragonfly 2024.1 (Object Research Systems, Montreal, QC, Canada).
- Avizo 2023.2 (Thermo Fisher Scientific, Waltham, MA, USA).

These software packages were used for sectioning, segmentation, 3D modelling, and preparation of atlas-standard figures according to chiropteran osteological conventions. Figures were prepared manually based on reconstructed slices and 3D models, following the orientation and atlas conventions commonly used in bat skull morphology.

### 2.5. Anatomical Terminology

Anatomical nomenclature follows the Nomina Anatomica Veterinaria [9] and the terminology standards used in chiropteran osteology [2,5,10].

**Figure 1:**
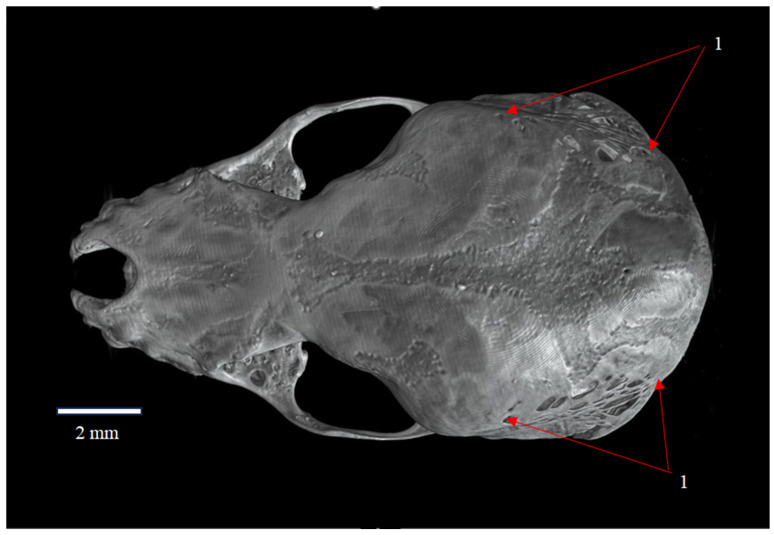
Dorsal view of the skull of *Plecotus auritus* (CZ-3). The posterolateral region of the parietal bone (ossa parietalia) shows the fenestrated surface termed Fenestras Elisabeth (nova structura), indicated by arrows (1).

**Figure 2:**
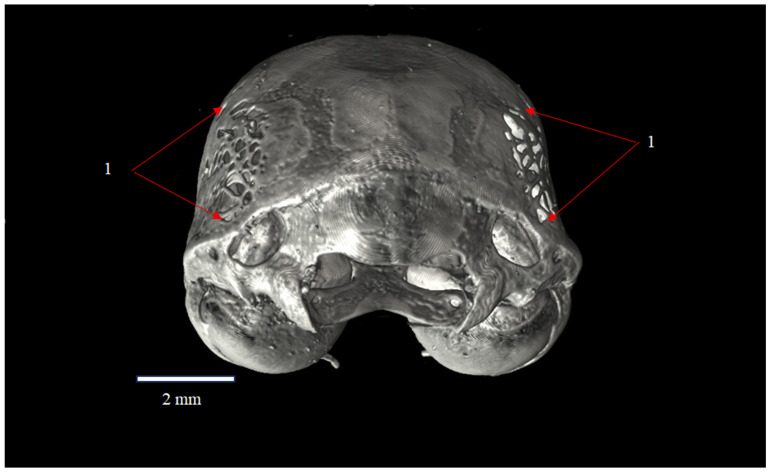
Occipital view of the skull of *Plecotus auritus* (CZ-3), showing the symmetry of fenestration (1) represented by the fenestrae parietales Elisabeth on the parietal bones (ossa parietalia). Red arrows indicate the location and extent of the fenestrated areas. The coloration reflects rendering settings and does not represent an artifact or red ware. Scale bar = 2 mm.

**Figure 3:**
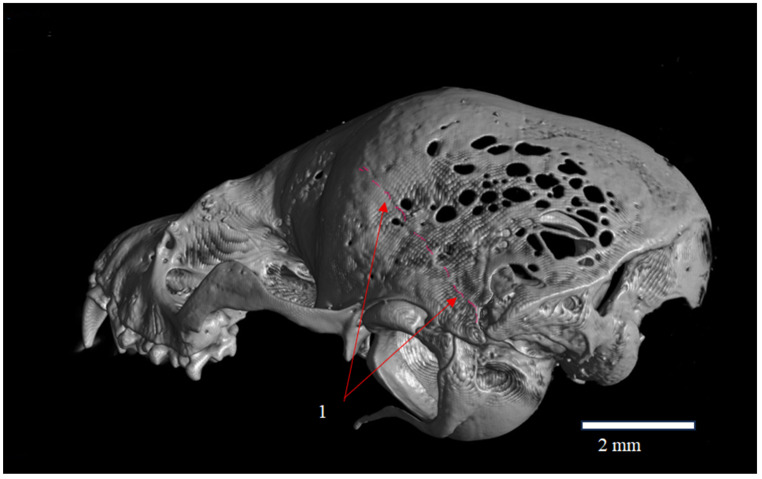
Posterolateral view of the skull of *Plecotus auritus* (CZ-3), showing the Fenestras Elisabeth surface and its topographic relationships with the temporal bone (os temporale) and its squamous part (pars squamosa). The arrow (1) indicates the topographic boundary of the Fenestras Elisabeth complex. Red arrows indicate the location and extent of the fenestrated areas. The red coloration represents graphical annotation only and does not correspond to red ware or any imaging artifact.

**Figure 4:**
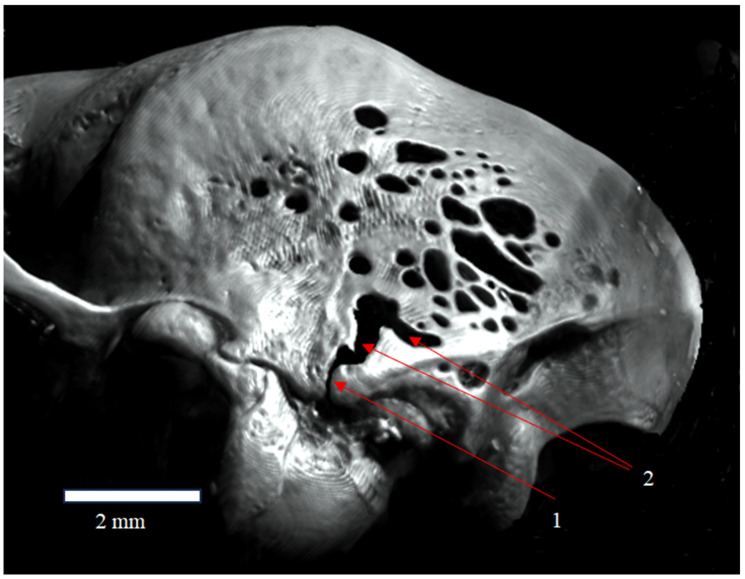
Posterolateral view of the skull of *Plecotus auritus* (CZ-2), showing (1) the occipitomastoid fissure (fissura occipitalis mastoidea) and (2) the fenestrula Elisabeth. Arrows indicate the location of the labelled structures.

**Figure 5:**
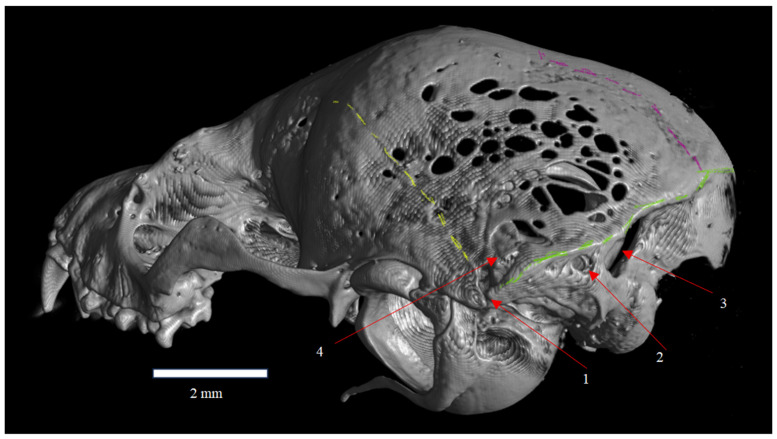
Posterolateral view of the skull of *Plecotus auritus* (CZ-3) showing color-marked boundaries of the cranial bones and the Fenestras Elisabeth surface on the parietal bone (os parietale). Highlighted bone boundaries: occipital bone (os occipitale), interparietal bone (os interparietale), and the squamous part (pars squamosa). Visible structures: (1) occipitomastoid fissure (fissura occipitalis mastoidea), (2) condylar canal (canalis condylaris), (3) external condylar canal (canalis condylaris externus), and (4) fenestrula Elisabeth. Different colors indicate the boundaries of individual cranial bones and serve as graphical annotations only; they do not represent imaging artifacts or material properties. Arrows indicate the location and extent of the labelled anatomical structures.

**Figure 6:**
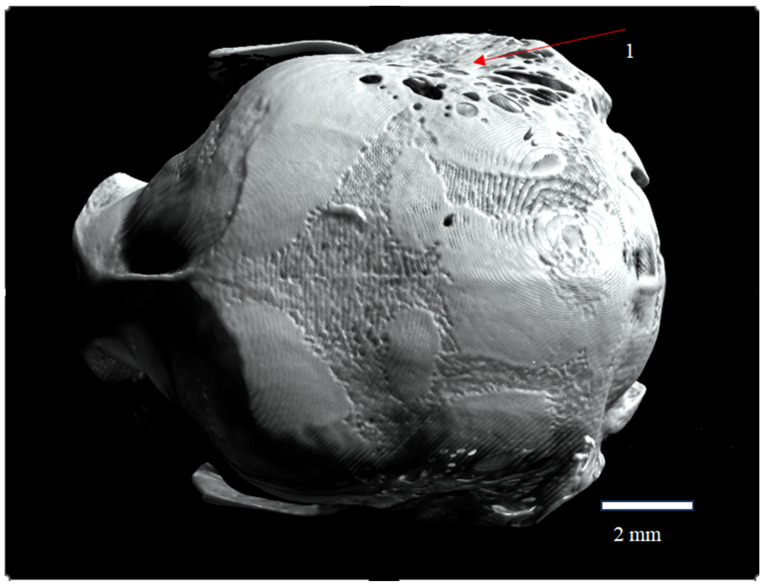
Dorsoposterior view of the skull of *Plecotus auritus* (CZ-2). A funnel-shaped central depression, identified as the acoustic collector (recessus acusticus parietalis), is visible within the Fenestras Elisabeth surface. The arrow indicates the location of the funnel-shaped central depression (recessus acusticus parietalis).

**Figure 7:**
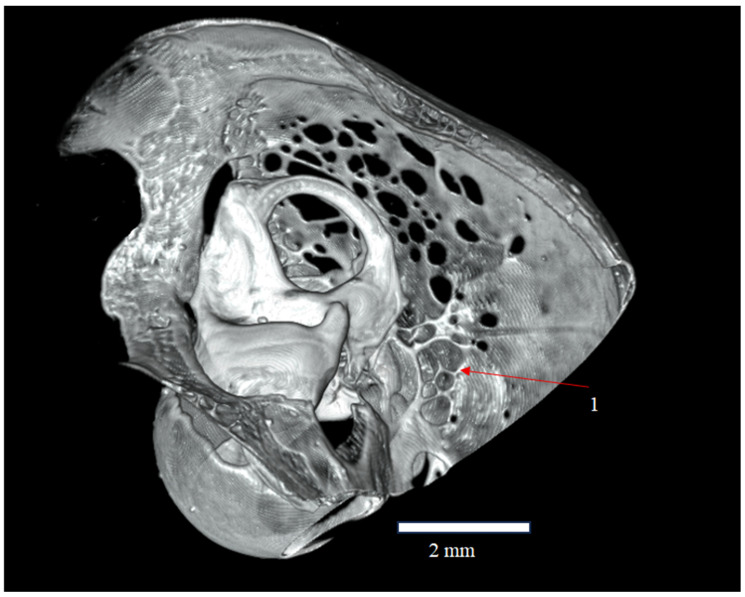
Sagittal section of the skull of *Plecotus auritus* (CZ-3), showing the semicircular canals (canales semicirculares) and the relationship of the Fenestras Elisabeth surface to the structures of the inner ear. The arrow and label (1) indicate a local bony continuity within the parietal region.

This study introduces and defines the fenestrated complex Fenestras Elisabeth, along with its constituent elements listed in Table 1 and illustrated in Figure 1, Figure 2, Figure 3, Figure 4, Figure 5, Figure 6 and Figure 7.

Newly identified morphological units (nova structura, nova structura topographica) include:

- Fenestra Elisabeth;
- fenestrae parietales Elisabeth;
- fenestrula Elisabeth dextra/sinistra;
- recessus acousticus parietalis;
- fissura occipitalis mastoidea.

Terminology is applied consistently throughout all descriptions, analyses, and figures.

## 3. Results

Micro-CT analyses provided high-quality three-dimensional reconstructions of the cranial vault of *Plecotus auritus*, allowing a detailed description of the newly recognised fenestrated complex of the parietal bone. The observations include both general features of the parietal surface and the structure, organisation, and variability of the elements forming the Fenestras Elisabeth complex (nova structura). Documentation is supplemented by a series of figures (Figure 1, Figure 2, Figure 3, Figure 4, Figure 5, Figure 6 and Figure 7) presenting the surface and sectional morphology of the analysed structures.

### 3.1. Overview of Parietal-Bone Surface Morphology

The parietal bone of *Plecotus auritus* exhibits a distinctly developed, thin-walled dorsolateral surface forming a large portion of the cranial vault. Within this region, extensive areas of fenestrated, lattice-like bone occur, corresponding to a tendency toward parietal fenestration reported in species of the genus *Plecotus*.

The external surface of the parietal bone is gently convex and smooth in posterior regions, whereas in the posterodorsolateral portion the bone undergoes marked reorganisation, gradually transitioning into the Fenestras Elisabeth structure (nova structura). This transition between smooth and fenestrated zones is gradual, with a clearly pronounced boundary formed by local thinning of the parietal lamina.

In axial and sagittal sections (Figure 7), notable variation in parietal thickness is apparent—the bone is thickest in the fenestration-free region and thinnest in the area of the fenestrated complex, especially near the temporal–parietal junction and the parietal–occipital line of contact. This gradient in thickness constitutes the structural basis for the development of the fenestrations described in the following subsections.

### 3.2. Fenestras Elisabeth Complex—General Description

The Fenestras Elisabeth complex (nova structura) represents an extensive fenestrated region located in the posterodorsolateral part of the parietal bone. The structure is defined by numerous openings (fenestrae) of variable size and shape (Figure 3, Figure 4, Figure 5 and Figure 6), which together form an organised fenestrated surface with clearly delineated topographic boundaries.

The complex occupies primarily the inferolateral portion of the parietal bone, beginning near the parietal–squamosal junction (pars squamosa) and extending dorsally to the parietal–occipital region (os occipitale). In the anteroposterior direction, the complex stretches from the dorsal portion of the occipital suture to the parieto-temporal suture and to the region directly adjacent to the posterior part of the squamosal crest.

Topographically, Fenestras Elisabeth displays a distinct pattern of openings, comprising both larger fenestrae and numerous smaller perforations distributed across the surface. The limits of the complex are marked by zones where fenestrations gradually disappear and the bone transitions into smooth parietal lamina. A key boundary is formed by the fissura occipitalis mastoidea (nova structura topographica), which establishes the inferior margin and separates the parietal region from the temporal region (Figure 3).

Morphologically, the complex is characterised by marked spatial variability in fenestration intensity—the largest number and size of openings occur in the lateral and posterodorsal regions, whereas toward the anterior and medial portions the fenestrations progressively diminish, forming a structural gradient. In sectional views (Figure 5, Figure 6 and Figure 7), a correlation is visible between the degree of fenestration and local thinning of the parietal bone.

The Fenestras Elisabeth complex comprises several distinct morphological elements (Table 1), among which the most important are: fenestrae parietales Elisabeth, fenestrula Elisabeth dextra et sinistra, and the recessus acousticus parietalis. These structures are described in detail in the subsequent subsections.

## 4. Structural Elements of the Complex

### 4.1. Fenestrae Parietales Elisabeth

The fenestrae parietales Elisabeth constitute the most numerous and most morphologically diverse elements of the Fenestras Elisabeth complex. These bony openings exhibit a wide range of morphologies, including oval, rounded, triangular, and irregular forms—and are distributed across the entire surface of the complex, with the highest density in its lateral and posterodorsal regions (Figure 3, Figure 4, Figure 5 and Figure 6).

Their diameter and shape show individual variability, although the overall mosaic-like arrangement remains consistent in all examined specimens. These fenestrations form the fundamental lattice-like framework of the complex, and their arrangement and orientation determine the course of local thickenings and bony bridges between adjacent openings.

In many areas, small fenestrae merge to form larger, irregularly outlined openings, suggesting dynamic remodelling characteristic of this region of the parietal bone. Each individual opening is referred to as an individual fenestra (fenestra parietalis Elisabeth) in accordance with the atlas nomenclature (Table 1).

### 4.2. Fenestrula Elisabeth Dextra et Sinistra

The fenestrula Elisabeth dextra and sinistra constitute a paired structural element of the Fenestras Elisabeth complex. These fenestrulae differ from the remaining fenestrations both in size and in their unusually regular shape. They are the largest oval openings of the complex, located symmetrically in its posteroinferolateral portion, directly adjacent to the occipitomastoid fissure (fissura occipitalis mastoidea) (Figure 3 and Figure 6). Each fenestrula is characterised by a distinctly outlined, smooth bony rim and a consistent oval contour. This combination clearly distinguishes them from the smaller and more irregular fenestrae parietales Elisabeth. Their symmetrical placement and constant presence in all analysed skulls indicate suggest a high degree of morphological conservatism within the species. Topographically, the fenestrulae form the boundary zone of the complex—their inferior margin remains in direct contact with the line of the fissura occipitalis mastoidea, making them an important reference point for identifying the structure. Due to their size, regularity and consistent spatial arrangement, the fenestrula Elisabeth are considered key morphological elements of the Fenestras Elisabeth complex and exhibit the highest diagnostic potential within the fenestrated system (Table 1).

### 4.3. Recessus Acusticus Parietalis

The recessus acousticus parietalis (nova structura) constitutes a clearly defined central depression within the Fenestras Elisabeth complex. It is the largest continuous surface within the fenestrated complex, forming a delicate, funnel-shaped bony depression surrounded by a ring-like distribution of fenestrae parietales Elisabeth (Figure 6). This depression is especially visible in sagittal and axial sections (Figure 5, Figure 6 and Figure 7), where it appears as a localized reduction in bone thickness consistently observed in all examined skulls. Its position corresponds to the course of structures of the inner-ear region, suggesting a potential acoustic and mechanical role. Within the recessus acusticus parietalis, the fewest fenestrations are observed, with larger openings occurring mainly around its periphery, while the central surface remains more compact and smooth. This arrangement forms a characteristic morphological pattern interpreted as a potential local reinforcement of the bone within the intensely fenestrated region of the complex.

The structure is present in all examined specimens and exhibits a high degree of repeatability, suggesting its taxonomic value and usefulness as a reference point in analysis of the Fenestras Elisabeth complex (Table 1).

### 4.4. Fissura Occipitalis Mastoidea

The fissura occipitalis mastoidea (nova structura topographica) is a distinct oblique fissure running along the boundary between the parietal bone (os parietale), the temporal bone (os temporale), and the occipital bone (os occipitale). This fissure defines the inferior and lateral limits of the Fenestras Elisabeth complex, separating the highly fenestrated region from the structurally denser portion of the skull.

Its course is morphologically stable and clearly visible both on the external surface (Figure 3) and in cross-sections (Figure 6), where it corresponds to a local thickening of the lamina. The structure serves as a consistent topographic boundary and is present in all analysed specimens.

### 4.5. Topographic Relationships

The topography of the Fenestras Elisabeth complex exhibits a high degree of inter-individual consistency. The greatest concentration of fenestrations occurs in the posterolateral region of the parietal bone (os parietale), within an area delineated by the following sutures:

- **sutura sagittalis**—dorsal boundary;
- **sutura lambdoidea**—posterodorsal boundary;
- **sutura squamosa**—lateral boundary (Figure 3, Figure 4, Figure 5 and Figure 6).

These sutures define a stable morphological framework within which the entire fenestrated complex is organized. Within this region, the fenestrations form a characteristic, fan-shaped arrangement extending from the parietal–occipital portion toward the anteroventral parietal zones.

In the immediate vicinity of the complex, within the squamous part of the temporal bone (pars squamosa ossis temporalis), small, isolated fenestration-like openings may be present. However, these are minute, irregular, and do not constitute a separate morphological unit. Their distribution appears incidental, and their overall contribution to the temporal surface is minimal. They do not show structural connections with the Fenestras Elisabeth complex and most likely represent secondary surface perforations.

The inferior boundary of the complex is formed by the fissura occipitalis mastoidea (nova structura topographica), an oblique fissure running between the parietal, temporal and occipital bones. In many specimens, the lower margin of the Fenestrula Elisabeth lies in direct contact with the line of this fissure (Figure 3 and Figure 6), indicating a close topographic association between the two structures.

In the posterolateral portion of the fenestrated complex, variation in the density and size of fenestrations is evident—small, numerous openings predominate in the posterodorsal region, while in the lateral and posteroventral areas larger fenestrae appear, including the Fenestrula Elisabeth. This forms a clear structural gradient visible both on the bone surface and in micro-CT sections (Figure 5, Figure 6 and Figure 7).

At the centre of the fenestrated complex lies the recessus acousticus parietalis, whose position corresponds to the course of the semicircular canal system (canales semicirculares) and elements of the inner ear. This suggests likely biomechanical and functional relevance of this depression in the context of species-specific adaptations.

The fenestration pattern is highly repeatable across specimens but not perfectly symmetrical. On the right side, numerous smaller openings tend to cluster, whereas on the left side larger and more elongated fenestrae predominate. Despite this individual variation, the overall spatial organisation of the complex remains consistent in all analysed skulls.

### 4.6. Occurrence and Individual Variability

The Fenestras Elisabeth complex was consistently present in all analysed skulls of *Plecotus auritus* (*n =* 10). In every specimen, both the basic fenestrated surface and the characteristic component structures were observed: fenestrae parietales Elisabeth, Fenestrula Elisabeth, and the recessus acousticus parietalis, together with the clearly defined fissura occipitalis mastoidea.

The stability of these structures indicates their conserved, species-level character.

At the same time, the fenestrations display pronounced individual variability in number, size and detailed distribution of openings. In some specimens, numerous small fenestrae (<1 mm) dominate, forming a dense mesh-like pattern. In others, larger and more elongated fenestrations appear, sometimes merging into individual elliptical or irregular windows (Figure 3, Figure 4, Figure 5 and Figure 6).

This variability does not, however, affect the overall topographic organisation of the complex:

- fenestrae parietales Elisabeth always occupy the posterolateral region of the parietal bone;
- the Fenestrula Elisabeth consistently lie in the posteroinferolateral part of the complex;
- the recessus acousticus parietalis is centrally located;
- the fissura occipitalis mastoidea defines the inferior boundary.

In most specimens, a slight asymmetry in fenestration between the right and left sides was recorded. Differences include the number of fenestrae, their clustering and the presence of one or two larger fenestrations on one side. This asymmetry falls within the natural range of individual variation and does not disturb the overall structural layout of the complex.

In summary, Fenestras Elisabeth is characterised by a stable, repeatable macroscopic arrangement combined with variability in fenestration microarchitecture. It represents a combination of diagnostic features—topographically stable but morphologically flexible—typical of anatomical units shaped by ongoing bone remodelling.

### 4.7. Summary of Results

Micro-CT analysis demonstrated that the posterolateral portion of the parietal bone in *Plecotus auritus* forms a clearly defined fenestrated complex, designated here as Fenestras Elisabeth (nova structura).

The complex is characterised by a well-organised architecture, repeatable topography and the presence of stable component elements, including: numerous fenestrae parietales Elisabeth, the paired Fenestrula Elisabeth, the centrally positioned recessus acousticus parietalis, and the distinct inferior boundary marked by the fissura occipitalis mastoidea.

Despite detailed variation in the number and size of fenestrae, the overall layout of the complex remains preserved in all examined specimens. The consistent position of major structural elements and their repeatable spatial relationships indicate that Fenestras Elisabeth constitutes a stable anatomical trait of the species.

The complex represents a three-dimensional morphological unit with likely functional significance in *Plecotus auritus*, distinguishing this species within Vespertilionidae, in which no comparable fenestrated complex has been previously reported. The results obtained here form the basis for further interpretation of the morphological and functional significance of the complex, which is addressed in the following section.

## 5. Discussion

### 5.1. Parietal Bone Fenestration in the Context of Mammalian Cranial Morphology

Fenestration of the parietal bone, particularly within the posterolateral region of the cranial vault, is uncommon among mammals and has only rarely been documented in the comparative osteological literature. In this context, the consistent presence and conserved topographic organisation of the Fenestras Elisabeth complex in all examined skulls of *Plecotus auritus* indicate that this structure is unlikely to be incidental and instead represents a stable morphological feature of the species. The present study is intentionally descriptive and focuses on defining the morphology and repeatable topographic organization of a previously undescribed cranial structure. A comprehensive morphometric and statistical analysis of fenestration number, size distributions, asymmetry, and related quantitative parameters is planned as a separate follow-up study building upon the anatomical framework established here.

Fenestration of the cranial vault, particularly within the posterolateral parietal region, appears to be uncommon in mammals and is only sporadically documented in the comparative osteological literature. In most taxa, the parietal lamina is described as compact and perforation-free, consistent with a structural role in maintaining the integrity of the cranial vault. Classical osteological works indicate that variation in parietal bone thickness is most commonly associated with the course of sutures or the presence of sinuses, rather than extensive surface fenestration [2,4,11].

Previous mentions of perforations in this region have typically been incidental and lacked detailed analysis. Occasional atlases and descriptive accounts of bat cranial morphology depict small openings or local thinning in the posterior parietal region of selected taxa; however, these features have typically been mentioned only briefly and without an explicit functional framework. Adding such comparative documentation helps to place the present observations within a broader descriptive context [2]. Contemporary approaches to mammalian cranial development and function tend to focus on the general organisation of bones, sutures and sinuses, and only rarely address fine-scale fenestration of the parietal lamina.

In most small mammals (e.g., Eulipotyphla, Rodentia), the parietal bone remains uniform, and local thinning or “lightening” of the bone develops primarily through sinus pneumatisation or remodelling of bony ridges [7]. Against this background, the extensive posterolateral fenestration observed in *Plecotus auritus* represents a unique phenomenon—both in its extent and in the repeatable spatial organisation revealed by micro-CT.

The consistent occurrence and conserved topography of this complex distinguish it from incidental thinning or sinus-related remodelling described in other small mammals.

### 5.2. Fenestras Elisabeth as a New Morphological Unit

In the posterolateral region of the parietal bone of *Plecotus auritus*, we identified a repeating fenestrated surface, designated as Fenestras Elisabeth (nova structura). This structure comprises multiple fenestrations (fenestrae parietales Elisabeth) of varying size and shape arranged in a characteristic fan-like pattern. Its extent and clearly defined boundaries, marked by the sutura sagittalis, sutura lambdoidea, and sutura squamosa, confirm its stability as a species-level trait across all analysed skulls.

In the inferolateral portion of the complex, a pair of large oval fenestrations—Fenestrula Elisabeth dextra et sinistra (nova structura)—form a symmetrical and distinctive component, consistently contacting the course of the fissura occipitalis mastoidea (nova structura topographica), which defines the inferior boundary of the complex.

Centrally, the complex contains a funnel-shaped depression, the recessus acousticus parietalis (nova structura), surrounded by numerous fenestrae parietales Elisabeth. Together, these four elements form a coherent and repeatable fenestrated complex in the posterolateral parietal region of *Plecotus auritus*.

This interpretation is informed by comparative osteological studies, including classical skull descriptions [2,4] and recent high-resolution reconstructions within Vespertilionidae [5,12], which document localised openings or thinning in the posterolateral parietal region, although without recognising them as a coherent morphological unit.

### 5.3. Evolutionary and Anatomical Context

The family Vespertilionidae is known for considerable cranial variability stemming from trophic, echolocatory and ontogenetic strategies [2,5,7]. Studies of *Myotis myotis* [6] and *Vespertilio sinensis* [12] indicate that modifications in these species concern primarily the proportions of the braincase and rostrum, cranial thickness and the degree of pneumatisation, but do not result in extensive fenestration of the parietal bone comparable to that identified in *Plecotus auritus*.

Similarly, 3D analyses of *V. sinensis* revealed ontogenetic dynamics related to remodelling of parietal and masticatory structures, but no analogous fenestrated complex has been documented at any developmental stage. Even in juvenile *Myotis myotis*, variation concentrates on the distribution of sutures and the degree of frontal and parietal closure [12], without mention of parietal fenestration.

Thus, Fenestras Elisabeth in *Plecotus auritus* represents an unusual pattern of cranial organisation, in which a reduction in bone mass is associated with the development of multiple fenestrae, rather than with classical sinus pneumatisation.

### 5.4. Functional Significance of Fenestration—Biomechanical and Acoustic Interpretations

The complex arrangement of fenestrations within Fenestras Elisabeth suggests that the structure may serve a function more sophisticated than simple reduction in bone mass. The consistent position of fenestrae parietales Elisabeth, their concentration in the posterodorsolateral parietal region, and their close association with the fissura occipitalis mastoidea (the boundary of the occipitotemporal region) indicate that the fenestrations may participate in redistributing mechanical stresses and microstrains produced during movements of the large, mobile pinnae [13]. Species of *Plecotus* possess extremely elongated and highly mobile pinnae capable of significant rotational and flexional movement relative to the skull. The lightweight fenestrated parietal lamina may therefore represent a compensatory mechanism that locally reduces bone mass and dissipates mechanical energy as an elastic network of openings, while maintaining the structural integrity of the skull vault.

Surrounded by fenestrations, this depression may influence local vibrational behaviour of the parietal lamina and thus could potentially modulate mechanically transmitted microvibrations in the adjacent region [14]. Such an arrangement may enhance sound conduction while limiting resonance risks in species with extremely sensitive echolocation. These interpretations are necessarily hypothetical and are not directly tested by the present data but are proposed to provide a functional framework for future experimental and modelling studies.

A loose comparative reference can be found in owls (e.g., *Tyto alba*), where asymmetries of cranial and auditory structures are associated with enhanced sound localisation; although the underlying anatomical substrates and evolutionary contexts differ substantially, this comparison illustrates how cranial microarchitecture may influence acoustic processing of sound sources [15,16]. Although the underlying mechanisms differ, the direction of adaptation—fine-tuning the acoustic properties of the skull—may be functionally analogous.

In *Plecotus auritus*, with its highly developed echolocation and mobile pinnae, fenestration of the posterolateral parietal region may reflect a response to combined biomechanical and acoustic constraints, potentially convergent in function—though not in structure—with mechanisms described in Strigiformes.

### 5.5. Individual Variability and Adaptive Plasticity of Elisabeth’s Surface

Analysis of fenestration patterns within Fenestras Elisabeth revealed that this structure is simultaneously species-specific and individually variable. While present in all examined skulls of *Plecotus auritus*, the number, size and shape of fenestrae displayed clear individual differences. In some specimens, the surface was dominated by numerous small openings; in others, larger fenestrations prevailed, sometimes merging into composite windows.

Yet, the overall topographic organisation remained consistent.

Variability also affected the depth and extent of the recessus acousticus parietalis, whose contours ranged from shallow and wide to pronounced and steep-walled. Despite these differences, the topography of the complex remained unchanged: the fenestrations always occupied the posterolateral parietal region, and the boundaries defined by the sutura sagittalis, sutura lambdoidea, and sutura squamosa were conserved relative to the region of occurrence.

Asymmetric fenestration between the left and right sides was observed in many individuals, involving differences in fenestra number, clustering and the presence of larger fenestrae on one side. This asymmetry may be tentatively interpreted in a broader comparative context, as asymmetries of cranial and pinna structures have been widely reported in taxa relying on precise sound localisation [3,15,16,17]. In *Plecotus auritus*, such differences may reflect morphological plasticity of the acoustic system, enabling micro-regulation of sound reception and dispersion depending on signal direction and intensity.

This combination of features—stable topography, variation in fenestration number and shape, and lateral asymmetry—indicates that Fenestras Elisabeth may be a structure of high adaptive plasticity.

It may have evolved as an elastic morphological element capable of individual fine-tuning in response to differences in pinna mobility or cranial biomechanics, while preserving an overall species-specific organisational pattern.

## 6. Conclusions

1. In *Plecotus auritus*, we describe for the first time an extensive fenestrated complex in the posterolateral region of the parietal bone, designated as Fenestras Elisabeth (nova structura).
2. This structure represents a stable and repeatable morphological element not previously reported in the family Vespertilionidae.
3. Within the Fenestras Elisabeth complex, we identified a set of topographically integrated units, including:
  ◦ numerous fenestrae parietales Elisabeth;
  ◦ the paired Fenestrula Elisabeth dextra/sinistra (nova structura);
  ◦ the centrally positioned recessus acousticus parietalis (nova structura);
  ◦ and the fissura occipitalis mastoidea (nova structura topographica).

Together, these structures form a coherent and repeatable fenestrated system in the posterolateral parietal region.

The pattern, organisation and distribution of fenestrations—along with their close association with the inner-ear region—suggest the presence of dual biomechanical and acoustic constraints.

The Fenestras Elisabeth complex may be associated with local reduction in bone mass in the cranial vault, while its precise biomechanical or acoustic implications remain to be explored in future studies. Individual variability in the number and shape of fenestrae, lateral asymmetry, and the repeatable topographic layout indicate high adaptive plasticity of this structure.

The Fenestras Elisabeth complex may be discussed in a broad comparative context alongside asymmetric acoustic adaptations described in Strigiformes; however, any functional analogy remains speculative and requires targeted experimental validation.

## Figures and Tables

**Table 1:** Atlas entry and morphological definition of the Complexus Fenestras Elisabeth.

Latin Name	English Name	Type of Unit	Morphological Description and Topographic Relations	Figures/First Use
**Fenestra Elisabeth**	Elisabeth’s surface	*nova structura*	A broad, fenestrated surface located in the posterolateral region of the parietal bone (os parietale), comprising numerous openings of variable shape and size. This is the principal structure of the fenestrated complex diagnostic for *Plecotus auritus*.	Figure 3, Figure 4, Figure 5 and Figure 6; first described herein
**fenestrae parietales Elisabeth**	Elisabeth’s parietal openings	structural element	Numerous fenestrations of variable morphology (oval, triangular, irregular), distributed across the entire surface of Fenestra Elisabeth. Individual openings are referred to as an individual fenestra (fenestra parietalis Elisabeth).	Figure 3, Figure 4, Figure 5 and Figure 6
**Fenestrula Elisabeth dextra/sinistra**	Right/left Elisabeth’s window	nova structura	A pair of characteristic oval fenestrations located symmetrically in the inferolateral portion of Fenestra Elisabeth, directly adjacent to the occipitomastoid fissure (fissura occipitalis mastoidea). These openings exhibit a highly regular shape and consistent position in all analysed specimens.	Figure 3, Figure 4, Figure 5 and Figure 6; first described herein
**Recessus acousticus parietalis**	Parietal acoustic recess	nova structura	A funnel-shaped central depression within Fenestra Elisabeth, surrounded by the network of fenestrations. Its lower portion borders the inner-ear region, suggesting a likely acoustic role.	Figure 6; first described herein
**Fissura occipitalis mastoidea**	Occipitomastoid fissure	nova structura topographica	An oblique fissure separating the parietal bone from the squamous and mastoid portions of the temporal bone. It contacts the fenestrula Elisabeth, forming its lower topographic boundary.	Figure 3 and Figure 6; first described herein

## Data Availability

Data available on request from the corresponding author.
